# The Results of Gastric Surgery

**Published:** 1912-08-31

**Authors:** 


### The Hospital

The Workers' Newspaper of Administrative Medicine and Institutional Life, Administration, National Insurance and Health.

No. 1365, Vol. LII. SATURDAY, AUGUST 31, 1912. PRICE ONE PENNY.

### THE RESULTS OF GASTRIC SURGERY.

A kind of work which hospital registrars have exceptional facilities for carrying out is that of following up the after-history of discharged patients, with a view to testing the effects of a particular operation or other mode of treatment. Statistics thus obtained are of far more value than those collected by an individual consultant, for several reasons: one is that the element of individual bias is eliminated, since the statistician is not following up the results of his own operations, and is therefore much less tempted to take too roseate a view; another is that the practice of several operators is being collected, not merely that of some one who may have brought the technique of his favourite operation to a pitch of perfection not generally attainable; another, that the results of the operation as it affects the health of people who have to return often to life and work in not very hygienic conditions are shown forth; and, lastly, that it is possible to deal with larger numbers, and so to avoid some, at least, of the proverbial fallacies of statistics. That more frequent use is not made of the opportunities of this sort which hospital registrars enjoy is certainly regrettable. As a notable instance of well-doing in this respect, attention may be directed to a careful summary by Dr. A. R. Short of all the gastric cases treated in the surgical wards of the Bristol Royal Infirmary during the years 1902-10 inclusive.* Out of 165 patients on the registers, 75 were successfully traced, 30 were lost sight of, and 60 died. Of the 75 all but two were traced for more than six months, and many of them for several years. For full details of the various conditions treated, reference should be made to the original paper; but some of the general conclusions reached are of much interest and deserve remark.

The extraordinary value of gastro-enterostomy for simple ulcer in the stomach and duodenum is well illustrated. Thirty patients out of thirty-five thus operated upon and traced wrote to say that they were in perfect health and could eat and digest anything. Many of these patients had endured chronic misery and ill-health for years, and the enthusiasm with which they write of the change from distress and disability to robust health is very encouraging. Curiously, when this operation is begun in the expectation of finding an ulcer, but no ulcer is found (there were twelve such cases), there is a smaller prospect of relief from symptoms being obtained by gastro-enterostomy than when a definite ulcer exists; at the same time, quite a fair proportion of these patients are permanently relieved or cured by operation. Another point of interest is the association of gastric ulcer, or symptoms of gastric ulcer, with movable kidney. Thus in one case a patient who had had a movable kidney moored two years previously was admitted dying of perforated gastric ulcer. In another nephrorrhaphy was successful for some years, but ultimately a gastric ulcer became troublesome. In another, no relief was secured from nephrorrhaphy (twice performed), but cure was effected by gastro-enterostomy.

There were sixty-one operations for perforated gastric or duodenal ulcer, with thirty-three recoveries; twenty-three of these patients were traced. Out of four in whom gastro-enterostomy or some equivalent short-circuiting operation was performed, three are completely and permanently cured of symptoms, and the fourth, though still dyspeptic, can eat ordinary food. Of the remaining nineteen, on the other hand, in whom the operation was simple suture with or without an omental graft, only four express themselves as quite cured; eleven still suffer from indigestion or vomiting, and two more returned for gastro-enterostomy, which cured one and considerably improved the other. There were thirty-three cases of cancer in the list. Twenty times the abdomen was opened and closed again at once without further interference; only one of these twenty patients survived as long as four months, but he lived two and a half years. Eight times a gastro-jejunostomy was performed; two of the patients died of the operation and four obtained some transient relief from it. Four times only was partial gastrectomy carried out, and in two cases the operation proved fatal; the third patient died of recurrence in nine months, and the fourth was hale and hearty for two years, but since then has lost ground and is now proved to have recurrence. Such are the classified end-results of gastric surgery in the Bristol Royal Infirmary. They are well worth publishing, for they show both the triumphs (as in dealing with perforated gastric ulcer) and the limitations (as in the cancer cases) of modern surgery when serious affections of the stomach require relief. We trust the good example of Dr. Short will provoke emulation elsewhere among those who have the custody of similar mines of information.

* *Bristol Medico-Chirurgical Journal,* September 1911, p. 220.

